# Method Development and Validation of Almotriptan in Human Plasma by HPLC Tandem Mass Spectrometry: Application to Pharmacokinetic Study

**DOI:** 10.3797/scipharm.1112-01

**Published:** 2012-02-27

**Authors:** Konda Ravikumar, Babu Rao Chandu, Balasekhara Reddy Challa, Kottapalli B. Chandrasekhar

**Affiliations:** 1 Hindu college of Pharmacy, Amaravathi Road, Guntur, Andhrapradesh, 522002, India; 2 Jawaharlal Nehru Technological University, Anantapur, 515002, India; 3 Donbosco college of Pharmacy, Pulladigunta, Guntur, 522201, India; 4 Nirmala college of Pharmacy, Madras road, Kadapa, Andhrapradesh, 516002, India

**Keywords:** HPLC, MS/MS, Almotriptan, Human plasma, Pharmacokinetic study, LLE

## Abstract

A simple, sensitive and selective method has been developed for quantification of Almotriptan (AL) in human plasma using Almotriptan-*d*_6_ (ALD6) as an internal standard. Almotriptan and Almotriptan-*d*_6_ were detected with proton adducts at m/z 336.1→201.1 and 342.2→207.2 in multiple reaction monitoring (MRM) positive mode, respectively. The method was linear over a concentration range of 0.5–150.0 ng/mL. The limit of detection (LOD) and limit of quantification (LOQ) for Almotriptan were 0.2 pg/mL and 0.5 ng/mL, respectively. Liquid-liquid extraction was used followed by MS/MS (ion spray). The method was shown to be precise with an average within-run and between-run variation of 0.68 to 2.78% and 0.57 to 0.86%, respectively. The average within-run and between-run accuracy of the method throughout its linear range was 98.94 to 102.64% and 99.43 to 101.44%, respectively. The mean recovery of drug and internal standard from human plasma was 92.12 ± 4.32% and 89.62 ± 6.32%. It can be applied for clinical and pharmacokinetic studies.

## Introduction

**Fig. 1.**
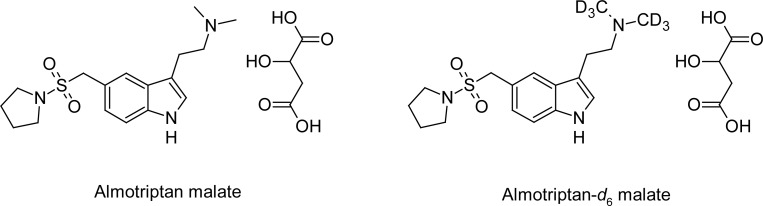
Chemical structure of Almotriptan malate and Almotriptan-*d*_6_ malate

Almotriptan, *N*,*N*-dimethyl-2-{5-[(pyrrolidin-1-ylsulfonyl)methyl]-1*H*-indol-3-yl}ethanamine, is a novel 5-HT1B/1D receptor agonist used for the treatment of symptomatic relief of migraines (Fig. 1) [1]. Almotriptan is absorbed well orally, with an absolute bioavailability of around 70%. The drug shows a dose linear pharmacokinetics and a mean elimination half-life of 1.4 to 3.8 h. Approximately 40 to 50 % of the dose is recovered unchanged in the urine; renal elimination probably occurs via active tubular secretion. The balance of the dose is eliminated unchanged in faecus (approximately 5%) or is metabolised [2, 3].

To our knowledge, several methods for the determination of Almotriptan in biological matrixes [1, 4, 5], pharmaceutical formulations [6–9] by LC–MS/MS [4], HPLC [6, 7] HPTLC [8], fluorimetric and calorimetry [9] have been reported.

However, Fleischhacker et al. [4] concentrated more on pharmacokinetics rather than method development and validation. The authors have not explained briefly extraction procedure, stability aspects, matrix factor effect, and recovery for determination of Almotriptan by LC-MS/MS. The purpose of this study was to develop and validate a novel sensitive LC-MS/MS method to quantify Almotriptan in human plasma.

## Material and methods

### Standards and chemicals

Almotriptan malate was obtained from USP and Almotriptan malate-*d*_6_ was obtained from clear synth Labs (P) Ltd, Mumbai, India. All other chemicals (Ammonium formate, formic acid, sodium carbonate, acetonitrile, methanol) and solvents were purchased from s. d. fine chemical’s Mumbai. Human plasma was obtained from Navjeevan blood bank, hyderabad, India.

### Instrumentation

Almotriptan was analyzed using HPLC system (1200 Series Agilent Technologies, Germany). MS/MS (ABI-SCIEX, Toronto, Canada) using MRM. A turbo electrospray interface in positive ionization mode was used. Data processing was performed on Analyst 1.4.1 software package (SCIEX).

### Detection

**Fig. 2.**
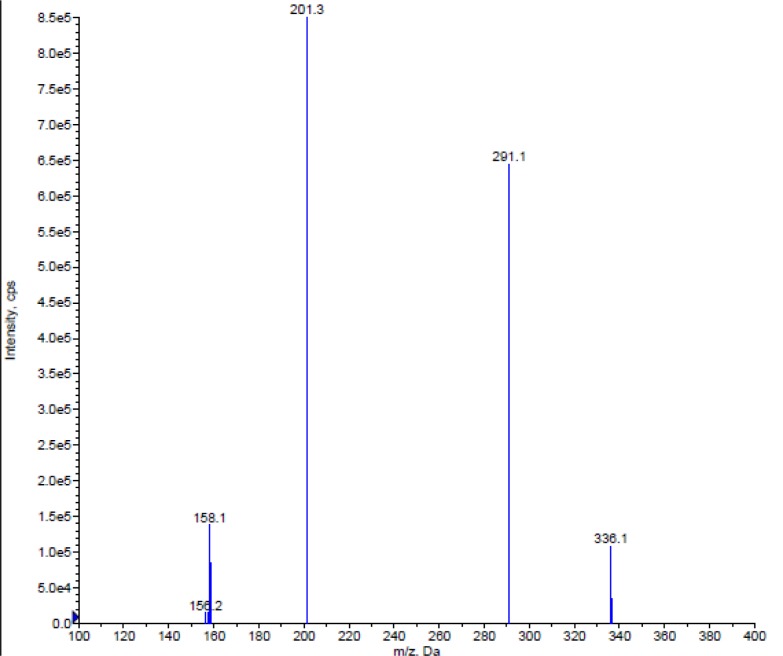
Mass spectra of the Almotriptan Q1, Almotriptan Q3

**Fig. 3.**
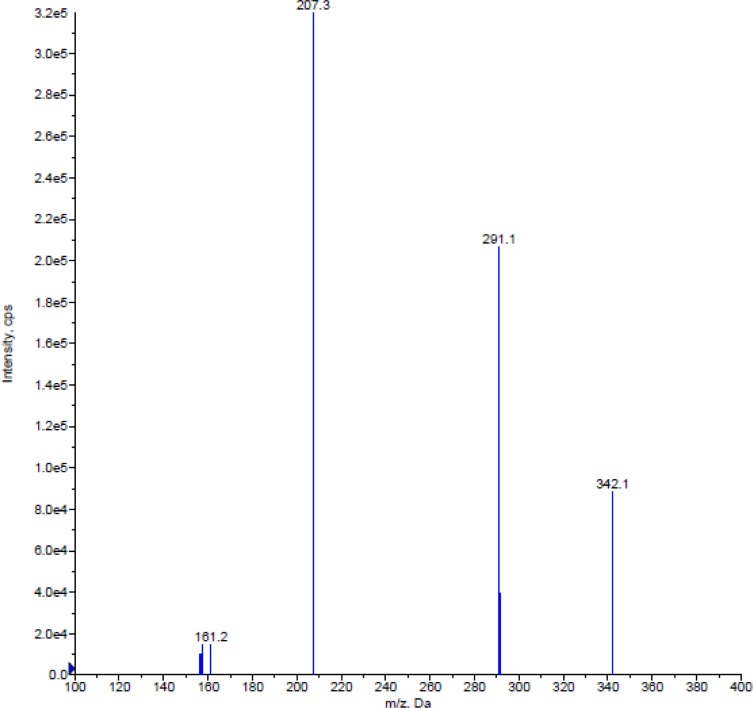
Mass-spectra of Almotriptan-*d*_6_ (Q1), Almotriptan-*d*_6_ (Q3)

Turbo Ion Spray (API) positive mode with Unit Resolution, MRM was used for the detection. For Almotriptan, the MH^+^ (m/z: 336.1) was monitored as the precursor ion and a fragment at m/z: 201.3 was chosen as the product ion (Fig. 2). For internal standard, the MH^+^ (m/z: 342.2) was monitored as the precursor ion and a fragment at m/z: 207.2 was monitored as the product ion (Fig. 3). Mass parameters were optimised as Source temperature 500 °C, Ion source gas 1 (GS1) 25 (nitrogen) psi, Ion source gas 1 (GS2) 35 (nitrogen) psi, Curtain gas 25 (nitrogen) psi, CAD gas 8 (nitrogen) psi, Ion Spray (IS) voltage 4000 volts, Source flow rate 500 μl/min without split, Entrance potential 10 V, Declustering potential 40 V for both analyte and IS, Collision energy 22 V for both Analyte and IS, Collision cell exit potential, 12 V for both Analyte and IS.

### Chromatographic conditions

Chromatographic separation was carried out on a reversed phase Zorbax, SB C18, 4.6 × 75mm, 3.5 μm column using a mixture of 10 mM ammonium formate buffer (pH 4.5) and acetonitrile (50:50 v/v) as mobile phase with a flow-rate of 0.5 mL/min. The column temperature was set to 40°C. Retention time of Almotriptan and Almotriptan-*d*_6_ was found at 1.5 ± 0.2 min approximately with a total runtime of 3 min.

### Preparation of standards and quality control (QC) Samples

To prepare stock standard solution (100 μg/mL) of Almotriptan, accurate volume of Almotriptan was dissolved in methanol in 20 ml volumetric flask. The stock solution was then further diluted with blank plasma to obtain the different working solutions ranging from 50, 150 and 1000 ng/mL, from which analytical standards were prepared at concentration levels of 0.5, 1.0, 5.0, 15.0, 30.0, 45.0, 60.0, 90.0, 120.0 and 150.0 ng/mL by appropriate dilution with blank plasma. Quality control (QC) samples were prepared at Lower limit of quality control (LLOQ) (0.5 ng/mL), Low quality control (LQC) (1.5 ng/mL), medium quality control (MQC) (75.0 ng/mL) and high quality control (HQC) (105.0 ng/mL) concentrations in the same way as the plasma samples for calibration. All samples were stored in a −30°C freezer until analysis.

### Sample preparation

Liquid-Liquid extraction procedure was used in this study to isolate Almotriptan from the plasma samples. For this purpose, 100 μL of Almotriptan-*d*_6_ (80 ng/mL) and 200 μL plasma (respective concentration of plasma sample) was added into riavials then vortexed for 30 sec and then 100 μl of 0.5 N sodium carbonate solution was added and vortexed for 10 min. Then samples were centrifuged at 4000 rpm for approximately 5 min at ambient temperature and the supernatant from each sample was transferred into respective ria vials, evaporated to dryness and reconstituted with 10mM ammonium formate (pH:4.5) acetonitrile (50:50v/v) and vortexed briefly. The sample was transferred into auto sampler vials to inject into LC-MS/MS.

### Linearity

Linearity was evaluated by using bulk spiked calibration curve standards and quality control standards. The calibration curve was constructed by using 10 non-zero calibration curve standard points spanning the range of 0.5–150.0 ng/mL, (0.5, 1.0, 5.0, 15.0, 30.0, 45.0, 60.0, 90.0, 120.0 and 150.0 ng/mL), four non-zero quality control standards (0.5, 1.5, 75.0 and 105.0 ng/mL), and, in addition, a blank sample (spiked only with blank plasma), blank + IS sample (spiked only with blank plasma and IS sample). Calibration curves were obtained by weighted 1/x^2^ linear regression model (y = mx + c). The ratio of Almotriptan peak area to Almotriptan-*d*_6_ peak area was plotted against the concentration of Almotriptan in ng/mL. The suitability of the calibration curve was confirmed by back-calculating the concentrations of the calibration standards.

### Precision and Accuracy

For determination of within-run and between-run precision and accuracy, four different series of samples at concentrations of 0.5, 1.5, 75.0 and 105.0 ng/mL of Almotriptan were analyzed within a single instrument run and in different runs. The accuracy was calculated from the ratio of measured concentration, based on the standard curve, to the nominal added concentration. Precision was evaluated by calculating the within-run and between-run coefficients of variations of the measured concentrations at each level (CV%).

### Recovery

The extraction recovery of Almotriptan and Almotriptan-*d*_6_ from human plasma was determined by analyzing quality control samples. Recovery at three concentrations (1.5, 75.0 and 105.0 ng/mL) was determined by comparing peak areas obtained from the plasma sample, and the standard solution was spiked with the blank plasma residue. A recovery of more than 50 % was considered adequate to obtain required sensitivity.

### Stability

Low quality control (1.50 ng/mL) and high quality control (105.0 ng/mL) samples (n=6) were retrieved from the deep freezer after three freeze-thaw cycles according to the clinical protocols. Samples were stored at −30°C in three cycles of 24, 36 and 48 h. In addition, the long-term stability of Almotriptan in quality control samples was also evaluated by analysis after 65 days of storage at −30°C. Autosampler stability was studied following a 57-h storage period in the autosampler tray with control concentrations. Bench top stability was studied for a 26-h period with control concentrations. Stability samples were processed and extracted along with the freshly spiked calibration curve standards. The precision and accuracy for the stability samples must be within ≤15 and ± 15 %, respectively, of their nominal concentrations [10].

### Application of method

The validated method has been successfully used to analyze Almotriptan concentrations in 18 human volunteers under fasting conditions after oral administration of a single tablet containing 12.5mg (1×12.5mg) Almotriptan. The study design was a randomized, two-period, two-sequence, two-treatment single dose, open label, bioequivalence study using AXERT^®^ (manufactured by Ortho-McNeil-Janssen Pharmaceuticals, Inc., USA) as the reference formulation. The test formulation was conducted for APL Research Pvt. Ltd, India. The study was conducted according to current GCP guidelines and after signed consent of the volunteers. Before conducting the study it was also approved by an authorized ethics committee. There was a total of 13 blood collection time points, including the predose sample, per period. The blood samples were collected at time intervals (0, 0.5, 1.0, 1.5, 2, 2.5, 3, 4, 6, 8, 12, 16 and 24 h) in separate vacutainers containing K_2_EDTA as an anticoagulant. The plasma from these samples was separated by centrifugation at 4000 rpm at 10°C. The plasma samples thus obtained were stored at −30°C until analysis. Post analysis, the pharmacokinetic parameters were computed using win nonlin® software version 5.2 and 90% confidence interval was computed using SAS® software version 9.2.

## Results and Discussion

### Method Development

The goal of this work was to develop and validate a simple, rapid and sensitive assay method for the quantitative determination of Almotriptan from human plasma samples by LC-MS/MS detection. We tested a wide spectrum of organic solvents from different physicochemical categories with different volume fractions as well as combinations. In terms of the analysis condition, various mobile phases, in different proportions, buffered and non-buffered at various pH, were attempted to provide the best peak shape and less retention time. Also, we tried different column packing, even from normal phase. The MS optimization was performed by direct infusion of solutions of both Almotriptan and Almotriptan-*d*_6_ into the ESI source of the mass spectrometer. The critical parameters in the ESI source include the needle (ESI) voltage. Other parameters, such as the nebulizer and the desolvation gases, were optimized to obtain a better spray shape, resulting in better ionization. A CAD product ion spectrum for Almotriptan and Almotriptan-*d*_6_ yielded high-abundance fragment ions at m/z 336.1→201.1 and 342.2→207.2 in multiple reaction monitoring (MRM) positive mode, respectively. After the MRM channels were tuned, the mobile phase was changed from an aqueous phase to a more organic phase with acid dopant to obtain a fast and selective LC method. The most accurate extraction method for analyte was selected as Liquid-Liquid extraction. A good separation and elution were achieved using 10 mM ammonium formate (pH 4.5.): acetonitrile (50:50 v/v) as the mobile phase, at a flow-rate of 0.5 mL/min and injection volume of 10 μL. The developed method was found to be the most sensitive and accurate one compared with known methods.

## Method Validation

### Linearity and Range

The method produced highly linear responses within the wide concentration range of 0.5–150.0 ng/mL, which is desirable for the majority of PK studies on the drug (Table 1).

### Specificity and Selectivity

**Fig. 4.**
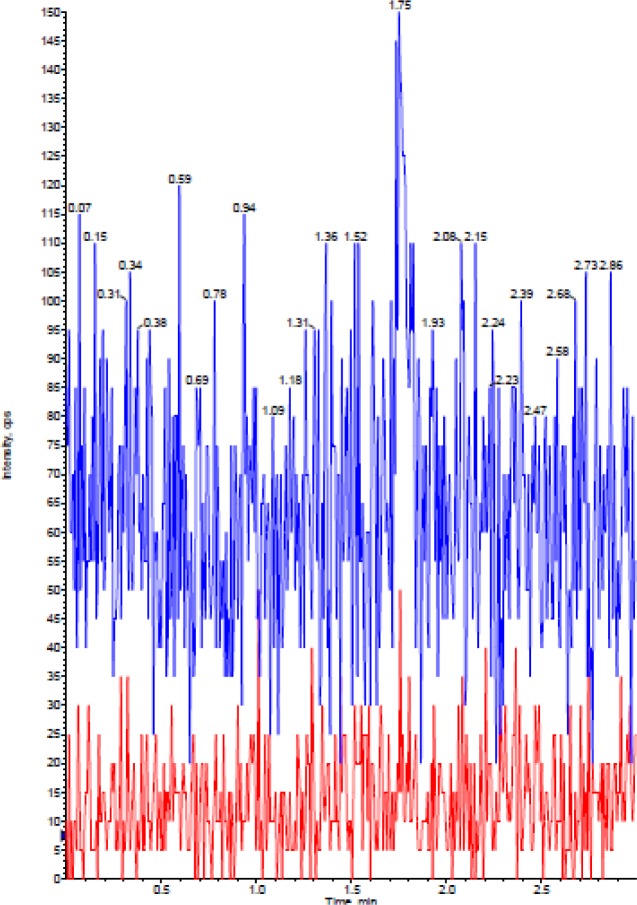
Chromatogram of Blank Plasma sample

**Fig. 5.**
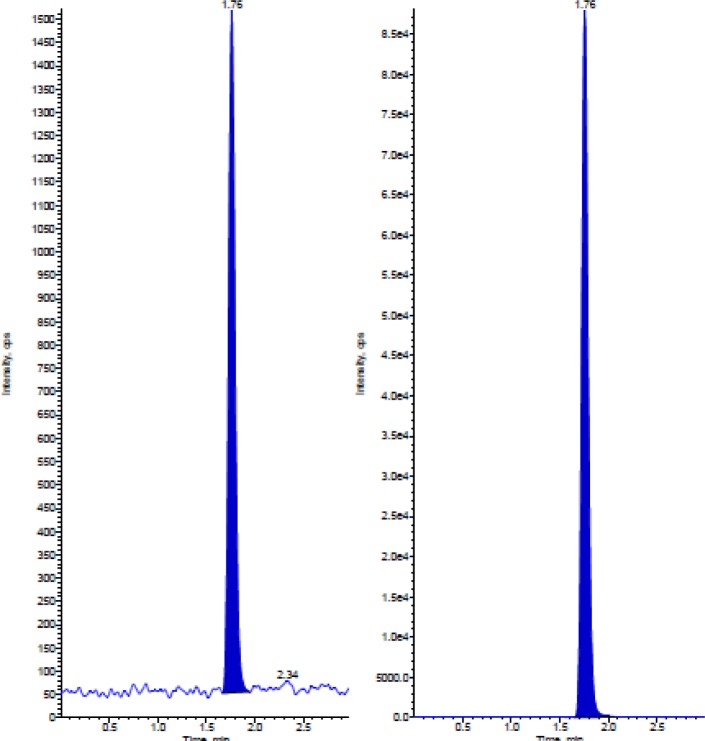
Blank human plasma spiked with 0.5 ng/mL Almotriptan and human plasma spiked with 100 ng/mL Almotriptan-*d*_6_ (LOQ)

To investigate specificity, a series of blank (drug-free) human plasma (total 6 plasma samples) in addition to the different concentrations spiked were screened, and no endogenous interference was observed at the retention time of Almotriptan and internal standard (Fig. 4 & 5).

### Precision and Accuracy

In Table 2, the CV% values of the measurements made by the method at different levels have been shown along with the corresponding accuracies. As shown, all the values of variations and accuracies are within the generally acceptable ranges (Precission (cv%) 15%, accuracy ± 15% for all concentrations, for LOQ accuracy ± 20%). This in turn assures obtaining accurate and precise results from the method.

### Recovery

A variety of extraction procedures were tested, as described, and the best recovery was achieved with Liquid-Liquid extraction. The mean recoveries of Almotriptan and Almotriptan-*d*_6_ were found to be 92.12 ± 4.32 % and 89.62 ± 6.32 %. These data indicate an acceptable degree of drug recovery by the extraction method within the whole concentration range tested.

### LOD and LOQ

**Fig. 6.**
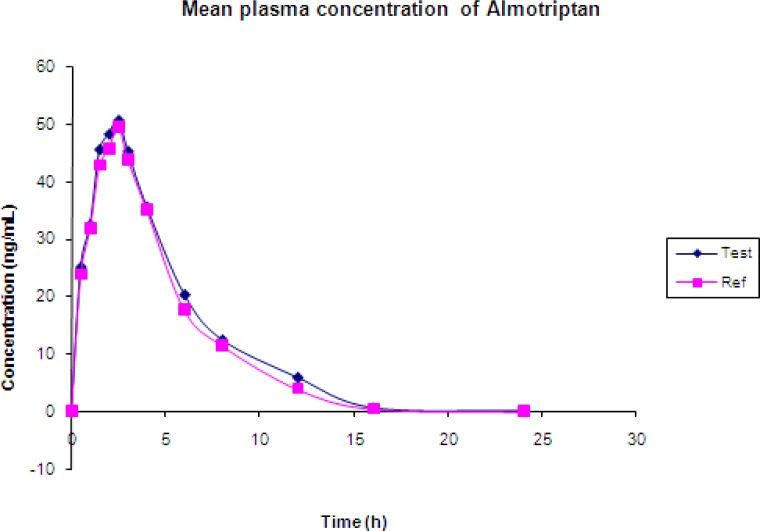
Mean Pharmacokinetic graph of Almotriptan in 18 human volunteers

The LOD and LOQ of the method for Almotriptan were 0.02 pg/mL and 0.50 ng/mL, respectively. These results confirm the significant sensitivity of the method for drug analysis (Fig. 6).

### Stability

Quantification of the Almotriptan in plasma subjected to 3 freeze-thaw (−30°C to room temperature) cycles showed the stability of the analyte. No significant degradation of the Almotriptan was observed even after the 57-h storage period in the autosampler tray. In addition, the long-term stability of Almotriptan in QC samples after 65 days of storage at −30°C was also evaluated. These results confirmed the stability of Almotriptan in human plasma for at least 65 days at −30°C (Table 3).

## Application

The validated method has been successfully used to quantify Almotriptan concentrations in 18 human volunteers, under fasting conditions after oral administration of 12.5 mg (1×12.5mg) tablet containing Almotriptan. The study was carried out after obtaining signed consent from the volunteers. These volunteers were contracted in APL Research centre, Hyderabad, India. The study protocol was approved from an IEC (institutional ethics committee) as per DCGI (Drug control general of India) guidelines. The pharmacokinetic parameters evaluated were Cmax (maximum observed drug concentration during the study), AUC_0–24_ (area under the plasma concentration–time curve measured 24 h, using the trapezoidal rule), *T*max (time to observe maximum drug concentration), Kel (apparent first-order terminal rate constant calculated from a semi-log plot of the plasma concentration versus time curve, using the method of the least square regression) and *T*1/2 (terminal half-life as determined by the quotient 0.693/Kel) (Table 4).

The 90% confidence intervals of the ratios of means Cmax, AUC0-24 within the acceptance range of 80–125%, (Table 5) demonstrate the bioequivalence of the two formulations of Almotriptan [11, 12]. The mean concentration versus time profile of Almotriptan in human plasma from 18 subjects that are receiving 1×12.5mg oral dose of Almotriptan tablet as test and reference is shown in Fig. 6.

## Conclusion

A simple, sensitive, rapid LC-MS/MS method with Liquid-Liquid extraction method was developed and validated as per FDA guidelines for quantification of Almotriptan in human plasma over a concentration range of 0.5–150.0 ng/mL. Almotriptan-*d*_6_ (ALD6) was used as an internal standard and 200 μL of plasma was used for extraction of drug and internal standard.

## Figures and Tables

**Tab. 1.** Calibration curves details

**Spiked plasma concentration (ng/mL)**	**Concentration measured (mean ± SD) (ng/mL)**	**CV (%) (n = 5)**	**Accuracy (%)**
0.50	0.49 ± 0.01	2.73	97.84
1.00	0.97 ± 0.01	1.37	96.84
5.00	4.89 ± 0.14	2.81	97.88
15.00	14.56 ± 0.22	1.52	97.09
30.00	29.22 ± 0.57	1.94	97.41
45.00	43.76 ± 0.74	1.70	97.24
60.00	58.49 ± 1.21	2.07	97.48
90.00	87.73 ± 1.81	2.07	97.48
120.00	117.25 ± 2.90	2.48	97.71
150.00	146.27 ± 3.10	2.12	97.51

**Tab. 2.** Precision and accuracy (analysis with spiked plasma samples at four different concentrations)

**Spiked plasma concentration (ng/mL)**	**Within-run**		
	**Concentration measured (*n*=6) (ng/mL) (mean ± SD)**	**% CV**	**% Accuracy**

0.50	0.51 ± 0.01	2.05	102.64
1.50	1.52 ± 0.04	2.78	101.00
75.00	74.20 ± 0.50	0.68	98.94
105.00	104.52 ± 0.49	0.47	99.54

**Spiked plasma concentration (ng/mL)**	**Between-run**		
	**Concentration measured (*n*=30) (ng/mL) (mean ± SD)**	**% CV**	**% Accuracy**

0.50	0.51 ± 0.01	0.86	101.44
1.50	1.50 ± 0.01	0.61	99.91
75.00	74.57 ± 0.42	0.57	99.43
105.00	104.21 ± 0.49	0.47	99.24

**Tab. 3.** Stability of the samples

**Spiked plasma concentration (ng/mL)**	**Room Temperature stability**		**Processed sample stability**	

	**26.0 h**		**57 h**	

	Concentration measured (n=6) (ng/mL) (mean ±SD)	% CV (*n*=6)	Concentration measured (n=6) (ng/mL) (mean ± SD)	% CV (*n*=6)
1.50	1.49 ± 0.13	2.6	1.51 ± 0.14	2.2
105.00	104.85 ± 1.20	3.2	104.37 ± 24.78	1.4

**Spiked plasma concentration (ng/mL)**	**Long term stability**		**Freeze and thaw stability**	

	**65 days**		**Cycle 3 (48 h)**	

	Concentration measured (n=6) (ng/mL) (mean ± SD)	% CV (*n*=6)	Concentration measured (n=6) (ng/mL) (mean ± SD)	% CV (*n*=6)
1.50	1.46 ± 0.04	0.7	1.49 ± 0.4	2.3
105.00	103.92 ± 6.13	1.2	104.81 ± 5.99	3.4

**Tab. 4.** Mean Pharmacokinetic Parameters of Almotriptan in 18 Healthy Volunteers after Oral Administration of 12.5 mg (1×12.5 mg) Test and Reference Product

**Pharmacokinetic Parameter**	**Almotriptan**	

	**Test**	**Reference**

AUC_0–t_ (ng h/mL)	293.55	272.24
Cmax (ng/mL)	50.76	49.75
AUC_0– ∞_ (ng h/mL)	293.55	272.24
Kel	0.34467	0.33982
Tmax (h)	2.5	2.5

AUC_0–∞:_ area under the curve extrapolated to infinity; AUC_0–t_: area under the curve up to the last sampling time; Cmax: the maximum plasma concentration; Tmax: the time to reach peak concentration; Kel: the apparent elimination rate constant.

**Tab. 5.** Test/Reference values for Log-Transformed Pharmacokinetic parameters of Almotriptan after Administration of 12.5 mg (1×12.5 mg) of Test and Reference products in 18 healthy male volunteers

**Pharmacokinetic parameters**	**Cmax**	**AUC_0−t_**	**AUC_0−∞_**
Test/Ref	102.02	107.83	107.82
